# Literature Review on the Effects of tDCS Coupled with Robotic Therapy in Post Stroke Upper Limb Rehabilitation

**DOI:** 10.3389/fnhum.2017.00268

**Published:** 2017-05-23

**Authors:** Davide Simonetti, Loredana Zollo, Stefano Milighetti, Sandra Miccinilli, Marco Bravi, Federico Ranieri, Giovanni Magrone, Eugenio Guglielmelli, Vincenzo Di Lazzaro, Silvia Sterzi

**Affiliations:** ^1^Research Unit of Biomedical Robotics and Biomicrosystems, Università Campus Bio-Medico di RomaRome, Italy; ^2^Unit of Physical and Rehabilitation Medicine, Università Campus Bio-Medico di RomaRome, Italy; ^3^Unit of Neurology, Neurophysiology and Neurobiology, Università Campus Bio-Medico di RomaRome, Italy

**Keywords:** stroke, cerebrovascular accident (CVA), upper-limb, tDCS, neurorehabilitation, robot-aided therapy

## Abstract

Today neurological diseases such as stroke represent one of the leading cause of long-term disability. Many research efforts have been focused on designing new and effective rehabilitation strategies. In particular, robotic treatment for upper limb stroke rehabilitation has received significant attention due to its ability to provide high-intensity and repetitive movement therapy with less effort than traditional methods. In addition, the development of non-invasive brain stimulation techniques such as transcranial Direct Current Stimulation (tDCS) has also demonstrated the capability of modulating brain excitability thus increasing motor performance. The combination of these two methods is expected to enhance functional and motor recovery after stroke; to this purpose, the current trends in this research field are presented and discussed through an in-depth analysis of the state-of-the-art. The heterogeneity and the restricted number of collected studies make difficult to perform a systematic review. However, the literature analysis of the published data seems to demonstrate that the association of tDCS with robotic training has the same clinical gain derived from robotic therapy alone. Future studies should investigate combined approach tailored to the individual patient's characteristics, critically evaluating the brain areas to be targeted and the induced functional changes.

## Introduction

Stroke is one of the leading factors of morbidity and mortality worldwide (Warlow et al., 2001).

In Italy, stroke annual incidence varies between 175/100.000 and 360/100.000 in men and between 130/100.000 and 273/100.000 in women (Sacco et al., 2011). Further, still in Italy, a total of 196.000 individuals are affected by stroke each year, 80% are new episodes and 20% are relapses (Gensini, 2005).

Activities of daily living (ADLs) and human quality of life strongly depend on upper limb functioning (Franceschini et al., 2010). Therefore, one of the goals of post-stroke upper limb rehabilitation is to recover arm and hand functions, and enable the patients to perform ADLs independently.

It is shown in the literature that intensive as well as task-specific training can be very effective in upper limb rehabilitation treatments after stroke (Feys et al., 2004; Lo et al., 2010; Klamroth-Marganska et al., 2014); this training should be repetitive, challenging and functional for the patients. To this purpose, robotics represents a key enabling technology for addressing these requirements for a well-stratified group of stroke patients (i.e., moderate-to-severe subjects). Clinical studies, varying in design and methods, have examined the effect of robotic devices on upper-limb and lower-limb rehabilitation in a clinical setting (Prange et al., 2006; Brewer et al., 2007; Mehrholz et al., 2015). Moreover, in a multicenter randomized controlled trial on moderate-to-severe chronic stroke patients, robotic therapy resulted superior to usual care and not inferior to intensive conventional rehabilitation treatment in terms of recovery of upper limb motor function (Lo et al., 2010). In addition, using robotic devices allows delivering new therapy constraints to maximize the required movement pattern (Kwakkel et al., 2007). Therefore, it is possible to control task learning phase more easily with robots than with traditional therapeutic techniques, since robots allows patients to perform guided movements on predefined pathways and avoid possible uncontrolled movements (Kwakkel et al., 2007).

Despite the interesting advancements in this area, the type of therapy leading to optimal results remains controversial and elusive and patients are often left with considerable disability (Bastani and Jaberzadeh, 2012).

Recently, the application of non-invasive neuro-modulation strategies to counteract inter-hemispheric imbalance has been acquiring a growing interest in post-stroke rehabilitation (Duque et al., 2005; Hummel and Cohen, 2006; Bolognini et al., 2009; Kandel et al., 2012). The adjunct of non-invasive interventions, such as the electrical brain stimulation or magnetic brain stimulation (Di Lazzaro et al., 2016), might be used to speed-up and maximize the potential benefit of rehabilitation treatments. In particular, transcranial Direct Current Stimulation (tDCS) may play an important role in stroke recovery since its capability to modify cortical excitability and neural activity (Lefaucheur, 2016; Lefaucheur et al., 2017).

In fact, modulating the excitability of a targeted brain region non-invasively, can favor a normal balance in the interhemispheric interaction and, hence, facilitate the recovery of motor functions of the paretic limb (Kandel et al., 2012).

tDCS consists of applying low-intensity current (1–2 mA) between two or multiple small electrodes on the scalp (Dmochowski et al., 2011). Depending on the electrode polarity, an opposite polarization of brain tissues can be induced with consequent modification of the resting membrane potential. Anodal stimulation will induce depolarization and increased cortical excitability; cathodal stimulation will induce hyperpolarization and decreased cortical excitability (Nitsche and Paulus, 2000; Fregni et al., 2005).

In the past, several studies have demonstrated a tDCS effect in terms of increased primary motor cortex activation assessed with fMRI (Hummel et al., 2005; Lindenberg et al., 2010).

The inter-hemispheric inhibitory competition model (Duque et al., 2005) implies that, to restore the interhemispheric balance altered after a stroke, one can either increase the excitability of the affected hemisphere with the anodal tDCS, or decrease the activity of the healthy hemisphere with cathodal tDCS (Hummel and Cohen, 2006).

The use of bilateral tDCS (applying simultaneously anodal electrode on the affected hemisphere and cathodal electrode on the unaffected hemisphere, Tazoe et al., 2014) could also be an effective strategy to produce interhemispheric rebalancing effects. Notwithstanding the promising achievements, the debate on tDCS efficacy in neurorehabilitation is still active and not entirely examined (Stagg and Johansen-Berg, 2013).

The application of tDCS might also have an impact on shoulder abduction (SABD) loading effects in individuals with moderate to severe chronic stroke; however, it is insufficient to make significant changes at higher SABD loads (Yao et al., 2015).

Furthermore, several neuromodulatory protocols have been applied together with robotic gait training to induce cortical plasticity and promote motor recovery after stroke. Motor excitability induced by paired associative stimulation, i.e., repetitive transcranial magnetic stimulation (rTMS) and tDCS has shown to be a potential neuromodulatory adjuvant of walking rehabilitation in patients with chronic stroke (Jayaram and Stinear, 2009) although there was no evidence regarding the efficacy of these protocols with respect to the others.

On the other hand, robot-assisted repetition with electromechanical gait trainer (Hesse et al., 1997; Hesse and Uhlenbrock, 2000) improved gait performance and maintained functional recovery at follow-up even during the chronic phase of stroke (Peurala et al., 2005; Dias et al., 2007). This could be likely due to the gait-like movement that allowed patients to practice a complete gait cycle, achieving better symmetric and physiological walking (Dias et al., 2007).

In this context, the adjunct of tDCS (delivered over the lower extremity motor cortex) to robotic locomotor exercises showed the capability to enhance the effectiveness of robotic gait training in chronic stroke patients (Danzl et al., 2013).

Conversely, while administering tDCS did not produce any reverse effects on chronic stroke patients, on the other hand it seemed to have no additional effect on robot-assisted gait training (Geroin et al., 2011). This could be due to the peculiar neural organization of locomotion, which involves both cortical (motor cortex) and spinal (central pattern generators) control (Dietz, 2002; Geroin et al., 2011).

Recently, another study has supported the hypothesis that anodal tDCS combined with cathodal transcutaneous spinal direct current stimulation (tsDCS) may be useful to improve the effects of robotic gait training in chronic stroke (Picelli et al., 2015).

Finally, combination of tDCS and robotic training has shown a promising strategy for improving arm, hand and lower extremity motor functions in persons with incomplete spinal cord injury (Raithatha et al., 2016; Yozbatiran et al., 2016).

All these approaches justify the growing interest of the scientific community in the evaluation of the effects of upper limb robot-aided motor training coupled with tDCS in stroke, relying on the adjunct of tDCS to further enhance primary effects of motor recovery (Triccas et al., 2016).

This paper intends to carry out an in-depth study of the literature regarding the effects of the combined use of tDCS and RT on motor and functional recovery in post stroke subjects. Moreover, the expected added value provided by this work is to complete the current knowledge in the neurorehabilitation field, by critically evaluating and comparing (when possible) the available results as well as discussing inconsistencies and possible issues. As a final goal, indications for the development of future and more specific rehabilitation protocols tailored to subject's needs are provided.

The paper is structured as follows. In Section “Overview of the Main Studies on tDCS Coupled with Upper-Limb Robotic Treatment” an overview of clinical studies that analyze effects of tDCS combined with upper limb robotic therapy (RT) is reported.

Section “Discussion” presents a critical discussion of the presented studies aimed to assess the efficacy of this novel combined approach. Finally, Section “Conclusions and future perspectives” reports final considerations and future suggestions.

## Overview of the main studies on tDCS coupled with upper-limb robotic treatment

The study of the effects deriving from the coupled use of tDCS and RT represents a relatively young field of interest. In fact, the number of studies that have tried to investigate and prove the successful combination of these two techniques is limited.

A wide literature search updated to January 2017 has been conducted resorting to the main databases, such as Pubmed Central (PMC), Cochrane, Scopus, Google Scholar. The following keywords have been employed: tDCS AND stroke^*^ OR ictus OR hemiplegia^*^ AND robot^*^ OR robotic therapy^*^, upper-limb rehabilitation, brain stimulation techniques, neurorehabilitation, rehabilitation robotics. Studies have been included only when focused on the novel therapeutic approach based on tDCS combined with robotic upper limb therapy.

The following inclusion criteria have been utilized:

1. Be a single session clinical trial (i.e., compare pre-treatment and post-treatment performance) or controlled trial (i.e., clinical trial with a control group, either randomized or not).
2. Involve stroke patients.
3. Concern movement therapy with a robotic device.
4. Include transcranial Direct Current Stimulation (tDCS) as Non-Invasive Brain Stimulation Technique.
5. Focus on upper-limb motor control (and possibly functional abilities).
6. Use relevant motor control and functional ability outcome measures.
7. Be a full-length publication in a peer-reviewed journal.

To enable the most complete overview of the current literature, the search has not been limited by patient subgroups (i.e., acute, subacute, or chronic) or by language.

**Figure 1:**
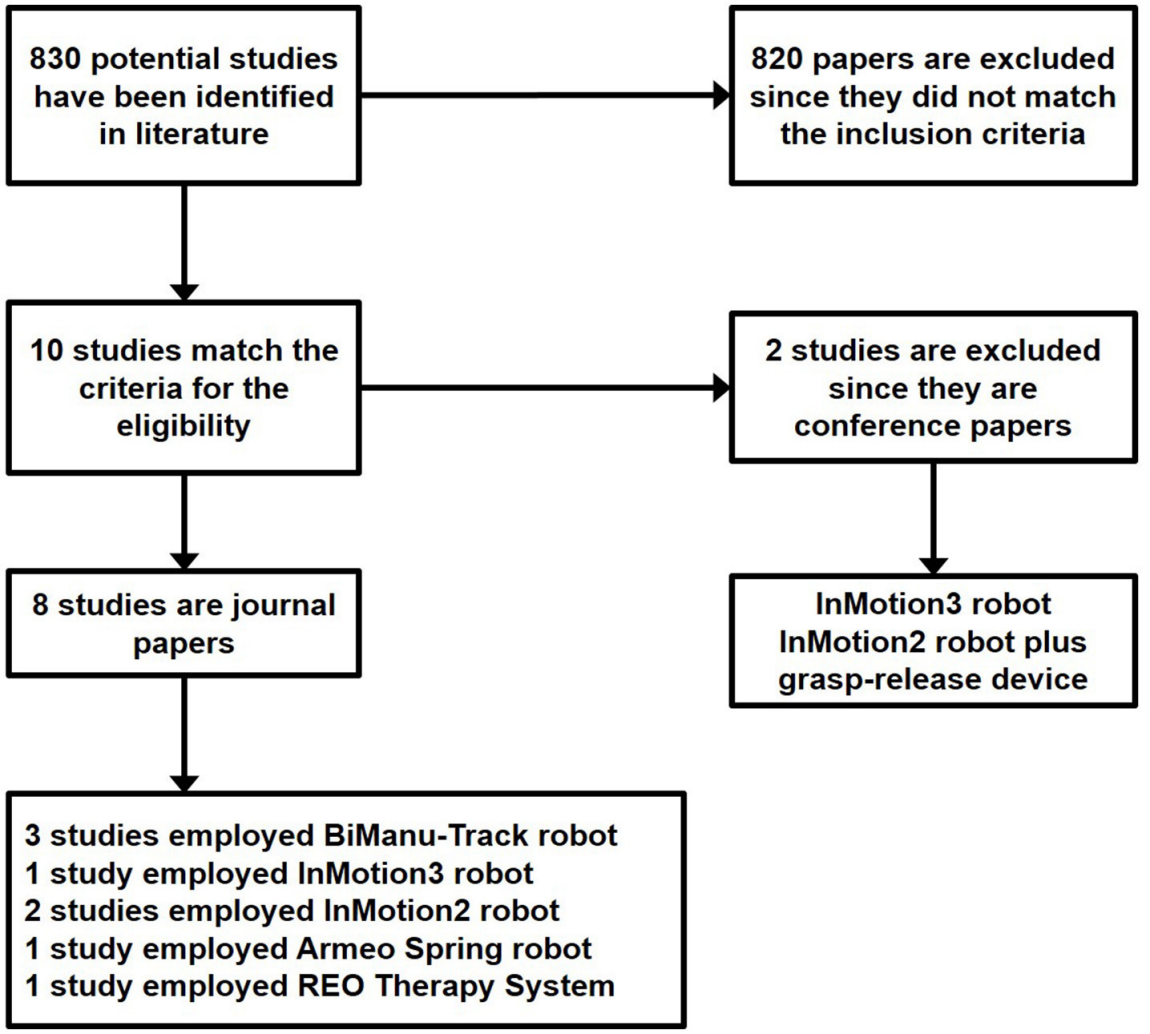
**Flowchart of the search and inclusion process**.

A flowchart of the search and inclusion process is shown in Figure 1. A total of 830 papers has been gathered by using the aforementioned search method. The abstracts matching the inclusion criteria have been selected. When appropriate, the full paper has been read. Therefore, from the initial 830 papers, 820 have been excluded since they did not meet the inclusion criteria. The remaining 10 papers have been carefully read. Eight studies are journal papers while 2 are conference papers.

The two conference papers Ang et al. (2012) and Mazzoleni et al. (2015) have not been included into the review analysis since they did not match the last inclusion criteria (i.e., be full-length publication in a peer-reviewed journal); however, their contents will be briefly discussed in the following.

The eight retrieved journal studies are Hesse et al. (2007), Hesse et al. (2011), Giacobbe et al. (2013), Ochi et al. (2013), Ang et al. (2015), Triccas et al. (2015), Powell et al. (2016), and Straudi et al. (2016).

The following contents have been extracted from the analysis of the selected studies:

1. Descriptive features of the subjects;
2. Protocol used within the study;
3. Outcome measures of motor control, functional abilities and neurophysiological parameters of cortical excitability;
4. Conclusions based on results.

The results of the studies are considered positive if the difference between pre-post treatment and between robot-trained and control groups is significant (*p* < 0.05) as calculated by an appropriate statistical tool.

The selected studies use different methods to evaluate the patients' recovery, including clinical scales, kinematics, and neurophysiological parameters of cortical excitability. The multiple outcome measures have been collected, as explained below:

1. Eight clinical scales regarding upper limb motor and functional assessment [Fugl-Meyer Scale (FM), Modified Ashworth Scale (MAS), Barthel Index (BI), Medical Research Council (MRC) score for muscle strength, Box and Block test (B&B), Motor Activity Log (MAL), Action Research Arm Test (ARAT), Stroke Impact Scale (SIS)];
2. Four neurophysiological parameters of motor cortical excitability [Motor Evoked Potential (MEP), Motor Imagery-Brain Computer Interface (MI-BCI) screening, motor map volume of ispilesional hemisphere, Center of Gravity (COG)];
3. Seven kinematic indices [Mean speed, peak speed, deviation, smoothness, duration, aim, and Hand Path Ratio (HPR, Dietz et al., 2011) in Cartesian space].

Notwithstanding, upper limb Fugl-Meyer score (FMS; Fugl-Meyer et al., 1974) has been chosen as the primary outcome measure for a comparative analysis, being the clinical tool adopted across almost all the studies.

All the current studies are grounded on the theory of interhemispheric competition where anodal tDCS is applied on the lesioned hemisphere (excitatory protocol) and cathodal tDCS is delivered on the contralateral hemisphere (inhibitory protocol). Sham stimulation is applied with an increasing current at training onset, and is switched off for the remainder of the stimulation time.

Although the selected studies share the general objective of assessing the effects of tDCS combined with the RT, the employed investigation methods are different and provide heterogeneous data that are difficult to be analyzed in a systematic way. However, despite the difficulty to find a global primary outcome measure, interesting common features have been extracted and a list of factors has been identified.

For the sake of clarity, the retrieved studies have been grouped in the following four categories while the main characteristics are reported in Tables 1A,B:

1. Effects of anodal and/or cathodal tDCS coupled with RT in post stroke patients (compared or not with RT alone);
2. Effects of different anodal tDCS delivering time, i.e., before, during or after RT;
3. Effects of anodal tDCS compared to cathodal tDCS when coupled with RT;
4. Effects of anodal tDCS combined with other neurostimulation techniques and RT.

**Table 1A:** **Overview of the studies on tDCS combined with robotic upper limb rehabilitation after stroke**.

**Study**	**Protocol therapy**	**Patients' number**	**Diagnosis**	**Age (Mean ± SD)**	**Time post-stroke**	**Description of the intervention**	**Kinematic indicators**	**Neurophysiological indicators**	**Clinical scales**
Hesse et al., 2007	Anodal tDCS +BiManuTrack RT	1 Group: 10 patients	Subacute ischemic stroke	63.3 years (SD is not directly reported)	4–8 weeks	20 min of RT with first 7 min of anodal tDCS (1.5 mA). 30 sessions (5 per 6 weeks)	–	–	FMS
Hesse et al., 2011	- Anodal tDCS + Bi ManuTrack RT - Cathodal tDCS + BiManuTrack RT - Sham tDCS + BiManuTrack RT	3 Groups: 32 patients for each group	Subacute ischemic stroke	Group A (real anodal): 63.9 ± 10.5 years Group B (real cathodal): 65.4 ± 8.6 years Group C (sham): 65.6 ± 10.3 years	3–8 weeks	Group A: 20 min of RT coupled with anodal tDCS (2.0 mA); Group B: 20 min of RT coupled with cathodal tDCS (2.0 mA); Group C: 20 min of RT coupled with SHAM tDCS. 30 sessions (5 per 6 weeks)	–	–	FMS, MAS, BI, MRC, B&B
Ochi et al., 2013	- Anodal tDCS + BiManuTrack RT - Cathodal tDCS + BiManuTrack RT	2 Groups: 9 patients for each group	Chronic: 11 hemorrhagic, 7 ischemic stroke	61.1± 10.0 years	(Mean) 4.4 years	1 mA anodal tDCS during first 10 min of RT; 1 mA cathodal tDCS during first 10 min of RT; 10 days, multiple sessions spaced out by 2-days rest	–	–	FMS, MAS, MAL
Giacobbe et al., 2013	Anodal Real/Sham tDCS+wrist RT (InMotion3)	1 group: 12 patients	Chronic ischemic stroke	64.4 ± 11.7 years	(mean) 4.0 years	TMS delivered for locating correct anodal position; - 20 min of wrist RT coupled with: - 20 min of REAL tDCS before RT; - 20 min of REAL tDCS during RT; - 20 min of REAL tDCS after RT; - 20 min of SHAM tDCS during RT. 1 single session	Cartesian space: Mean speed, Peak speed, Deviation, Smoothness, Duration, Aim	MEP (FCR, ECR)	FMS

*tDCS, Transcranial Direct Current Stimulation; RT, Robotic Training; TMS, Transcranial Magnetic Stimulation; MEP, Motor Evoked Potential; FMS, Fugl-Meyer Score; MAS, Modified Ashworth Scale; BI, Barthel Index; B&B, Box and Block Test; MRC, Medical Research Council; MAL, Motor Activity Log; FCR, Flexor Carpi Radialis; ECR, Extensor Carpi Radialis; SD, Standard Deviation*.

**Table 1B:** **Overview of the studies on tDCS combined with robotic upper limb rehabilitation after stroke**.

**Study**	**Protocol therapy**	**Patients' number**	**Diagnosis**	**Age (Mean ± SD)**	**Time post-stroke**	**Description of the intervention**	**Kinematic indicators**	**Neurophysiological indicators**	**Clinical scales**
Ang et al., 2015	- Bilateral Real/Sham tDCS + MI-BCI with robotic feedback (InMotion2)	2 Groups: 10 patients for real group, 9 patients for sham group	Chronic stroke: 13 ischemic 9 hemorrhagic; 18 subcortical 1 cortical	Group A (real): 52.1 ± 11.7 Group B (sham): 56.3 ± 9.5	9 months	Group A: 10 sessions of 20 min of bilateral tDCS (1 mA) before 1 h of MI-BCI with upper limb robotic feedback for 2 weeks. Group B: 10 sessions of 20 min of sham tDCS (1 mA) before 1 h of MI-BCI with upper limb robotic feedback for 2 weeks.	–	MI-BCI screening	FMS
Triccas et al., 2015	- Anodal Real/Sham tDCS+Armeo Spring RT	2 Groups: 12 patients for real group, 11 patients for sham group	12 Subacute: 10 ischemic and 2 haemorrhagic stroke; 11 Chronic: 8 ischemic and 3 haemorrhagic stroke	Group A (real): 64.3 ± 10 years Group B (sham): 62.5 ± 14.3 years	Subacute stroke: (mean) 8–10 weeks Chronic stroke: (mean) 3.1 years	Group A: anodal tDCS (1.0 mA) during first 20 min of 1 h RT; Group B: sham tDCS during first 20 min of 1 h RT; 18 sessions for 8 weeks (2–3 sessions per week)	Hand Path Ratio (HPR)	–	FMS, ARAT, MAL, SIS
Powell et al., 2016	- Anodal tDCS delivered before PNS or after PNS combined with robotic therapy (InMotion2)	2 Groups: 4 patients for tDCS before PNS, 6 patients for tDCS after PNS group	Chronic stroke: 7 ischemic 3 hemorrhagic; 6 left side lesion 4 right side lesion	Group A (before PNS): 61.0 ± 6.63 Group B (after PNS): 60.5 ± 2.95	4.5 years	Group A: 10 daily sessions of 20 min of anodal tDCS (2 mA) BEFORE 2 h of PNS followed by 2 h of RT Group B: 10 daily sessions of 20 min of anodal tDCS (2 mA) AFTER 2 h of PNS followed by 2 h of RT	–	Motor map volume of ipsilesional hemisphere, COG	FMS, SIS
Straudi et al., 2016	Anodal and cathodal Real/Sham tDCS+ReoGo Therapy System RT	2 Groups: 12 patients for real group, 11 patients for sham group	9 Subacute: 6 cortical and 3 subcortical (9 ischemic and 2 hemorrhagic), 14 Chronic: 8 cortical and 6 subcortical (10 ischemic and 2 hemorrhagic)	Group A (real): 52.7 ± 16 years Group B (sham): 64.3 ± 9.7 years	Subacute stroke: <6 months Chronic stroke: >6 months	Group A: 30 min of RT with anodal and cathodal real tDCS (1.0 mA); Group B: 30 min of RT with anodal and cathodal sham tDCS during first 30 s; 10 sessions for 2 weeks (5 sessions per week)	–	–	FMS, SIS

*tDCS, Transcranial Direct Current Stimulation; RT, Robotic Training; FMS, Fugl-Meyer Score; ARAT, Action Research Arm Test; SIS, Stroke Impact Scale; B&B, Box and Block Test; MAL, Motor Activity Log; MAS, Modified Ashworth Scale; MI, Motricity Index; MI-BCI, Motor Imagery—Brain Computer Interface; COG, Center of Gravity of the lesion; SD, Standard Deviation*.

The number of patients in the groups treated with real tDCS ranged from a minimum of 4 (Powell et al., 2016) to a maximum of 32 (Hesse et al., 2011) for a total of 148 patients; the control group has been used by Hesse et al. (2011) (32 patients), Triccas et al. (2015) (11 patients), Ang et al. (2015) (9 patients), Straudi et al. (2016) (11 patients). More details on the enrolled patients and the experimental groups are reported in Tables 1A,B. It can be observed that in the eight selected studies the enrolled patients vary for number, diagnosis (i.e., chronic or subacute stroke, cortical, or subcortical lesion) and group break down while the mean age is very similar.

Five different robotic devices have been used for delivering therapy across the analyzed studies: the RehaStim BiManu Track, used in three studies (Hesse et al., 2007, 2011; Ochi et al., 2013), the InMotion3 adopted in one study (Giacobbe et al., 2013), the InMotion 2 employed in two studies (Ang et al., 2015; Powell et al., 2016) the Armeo®Spring used by Triccas et al. (2015), and the REO Therapy System, Motorika, LTD, Israel used in Straudi et al. (2016).

For a detailed technical description of these devices please see, Hogan et al. (1993), Hesse et al. (2003), Sanchez et al. (2004), and Krebs et al. (2007).

### Effects of anodal and/or cathodal tDCS coupled with robotic treatment

Four studies have investigated the effects of tDCS combined with RT and/or compared the effects with RT alone.

The work in Hesse et al. (2007) was the first to investigate the feasibility of combining the two techniques with a single session pilot study. Results showed that subjects with subcortical lesion improved more than patients with cortical damage suggesting that the lesion site represents a determinant factor for evaluating treatment efficacy (Table 2A).

**Table 2A:** **Clinical scales scores**.

**Study**	**FMS (SD)**	**BI (SD)**	**MAS (SD)**	**MAL (SD)**	**MRC (SD)**	**B&B (*n* = 3)**
Hesse et al., 2007 Baseline	7.2 ± 3.1	–	–	–	3.0 ± 3.1	–
Post-treatment^*^	18.2 ± 17.2	–	–	–	7.6 ± 6.9	–
Hesse et al., 2011 Baseline	Group A tDCS(a): 7.81 ± 3.8 Group B tDCS(c): 7.9 ± 3.4 Group C tDCS(s): 8.2 ± 4.4	Group A tDCS(a): 34.1 ± 3.4 Group B tDCS(c):34.2 ± 7.6 Group C tDCS(s): 35.0 ± 7.8	Group A tDCS(a):1.6 ± 2.9 Group B tDCS(c):1.0 ± 1.8 Group C tDCS(s):1.4 ± 2.7	–	Group A tDCS(a): 3.5 ± 3.6 Group B tDCS(c):2.9 ± 3.4 Group C tDCS(s):3.4 ± 3.2	Group A tDCS(a): 0 Group B tDCS(c): 0 Group C tDCS(s): 0
Post treatment^**^	Group A tDCS(a): 19.1 ± 14.4 Group B tDCS(c): 18.9 ± 10.5 Group C tDCS(s):19.2 ± 15.0	Group A tDCS(a): 53.6 ± 14.5 Group B tDCS(c) 59.2 ± 12.4 Group C tDCS(s) 56.3 ± 15.5	Group A tDCS(a): 3.3 ± 3.6 Group B tDCS(c):3.5 ± 4.9 Group C tDCS(s):3.5 ± 4.0		Group A tDCS(a): 11.9 ± 12.5 Group B tDCS(c): 13.7 ± 10.4 Group C tDCS(s): 12.8 ± 12.1	Group A tDCS(a): 9 Group B tDCS(c): 8 Group C tDCS(s): 9
Ochi et al., 2013 Baseline	Group A tDCS(a): 23.2 ± 16.6 Group B tDCS(c): 23.6 ± 16.7	–	Group A tDCS(a): (E) 2.4 ± 1.1 (W) 3.0 ± 1.1; (F) 2.8 ± 1.3 Group B tDCS(c): (E) 2.5 ± 1.2 (W) 2.9 ± 1.1; (F) 2.9 ± 1.2	Group A tDCS(a): 1.6 ± 2.7 Group B tDCS(c): 1.6 ± 2.8	–	–
Post treatment^***^	Group A tDCS(a): 23.2 ± 16.6 Group B tDCS(c): 23.6 ± 16.7	–	Group A tDCS(a): (E) 2.4 ± 1.1 (W) 3.0 ± 1.1; (F) 2.8 ± 1.3 ^†^ Group B tDCS(c): (E) 2.5 ± 1.2 (W) 2.9 ± 1.1; (F) 2.9 ± 1.2 ^†^	Group A tDCS(a): 1.6 ± 2.7 Group B tDCS(c): 1.6 ± 2.8	–	–

*tDCS, Transcranial Direct Current Stimulation; RT, Robotic Training; FMS, Fugl-Meyer Score; MAS, Modified Ashworth Scale; BI, Barthel Index; B&B, Box and Block; MRC, Medical Research Council; MAL, Motor Activity Log; E, elbow; W, wrist; F, finger; SD, Standard Deviation*.

* *Significant difference occurred in FMS and MRC assessed between baseline and post treatment, p = 0.018 and p = 0.027, respectively*.

** *No between group differences occurred for all clinical indicators used (p > 0.025). Significant difference (p = 0.014) only occurred within the cathodal group (TACI+LACI vs. LACI) in terms of ΔFMS (not directly reported in Table 2A)*.

*** *Small but significant improvements (p < 0.05) between pre/post treatment, have been observed for both stimulation protocol in FMS and MAS (not in MAL, p > 0.05)*.

† *Between stimulation condition, i.e., tDCS(a) and tDCS(c), only for tDCS(c)+RT a significant improvement in MAS for the fingers has been observed*.

The other three works, Hesse et al. (2011), Triccas et al. (2015), and Straudi et al. (2016) performed randomized controlled trials with a larger sample of patients. However, they have shown that the adjunct of tDCS to RT only lead to small significant changes in FMS adjusting statistical analysis for the lesion site (cortical vs. subcortical), the timing from the stroke onset (chronic vs. subacute) and the type of stroke (ischemic vs. hemorrhagic) (Tables 2A–C).

**Table 2B:** **Clinical scales scores**.

**Study**	**FMS (SD)**	**MAL (SD)**	**B&B (*n* ≥ 3)**	**SIS (SD)**
Ang et al., 2015 Baseline	Group A real tDCS: 35.3 ± 7.8 Group B sham tDCS: 32.6 ± 8.1	–	–	–
Average improvement between post intervention and baseline^***^	Group A real tDCS: 0.9 ± 3.0 Group B sham tDCS: 2.8 ± 4.0	–	–	–
Triccas et al., 2015 Baseline	Group A real tDCS: 24.91 ± 16.01 Group B sham tDCS: 37.09 ± 13.57	–	–	–
Post treatment^****^	Group A real tDCS: 33.64 ± 16.25 Group B sham tDCS: 44.82 ± 16.29	–	–	–
Powell et al., 2016 Baseline	Group A tDCS pre-PNS: 23.3 ± 15.8 Group B tDCS post-PNS: 18.7 ± 8.1	–	–	Group A tDCS pre-PNS: 65.3 ± 5.1 Group B tDCS post-PNS: 60.0 ± 9.7
Average improvement between post intervention and baseline^*****^	Group A tDCS pre-PNS: 1.5 ± 1.39 Group B tDCS post-PNS: 0.17 ± 1.3	–	–	Group tDCS pre-PNS: 6.33 ± 2.21 Group B tDCS post-PNS: 0.50 ± 1.56
Straudi et al., 2016 Baseline	Group A real anodal/cathodal tDCS: 24.08 ± 16.60 Group B sham anodal/cathodal tDCS: 21.09 ± 13.19	Group A real tDCS (a+c) AOM: 0.68 ± 0.90 Real tDCS (a+c) QOM: 0.69 ± 1.01 Group B sham tDCS (a+c) AOM: 0.59 ± 1.02 Sham tDCS (a+c) QOM: 0.59 ± 1.17	Group A real anodal/cathodal tDCS: 10.42 ± 15.47 Group B sham anodal/cathodal tDCS: 6.55 ± 11.67	–
Post treatment^******^	Group A real anodal/cathodal tDCS: 28.50 ± 18.96^******^ Group B sham anodal/cathodal tDCS: 26.64 ± 16.12^******^	Group A real tDCS (a+c) AOM: 1.09 ± 1.36§ Real tDCS (a+c) QOM: 1.05 ± 1.43§ Group B sham tDCS (a+c) AOM: 0.89 ± 1.38 Sham tDCS (a+c) QOM: 0.85 ± 1.50	Group A real anodal/cathodal tDCS: 12.67 ± 17.23§ Group B sham anodal/cathodal tDCS: 8.55 ± 14.07	–

*tDCS, Transcranial Direct Current Stimulation; RT, Robotic Training; FMS, Fugl-Meyer Score; B&B, Box and Block; MAL, Motor Activity Log; MI, Motricity Index; CM, Chedoke Mc-Master Scale; UE, upper limb; AOM, Amount of Movement; QOM, Quality of Movement; SD, Standard Deviation*.

*** *Significant difference occurred in FMS (UE) and MI assessed between baseline and post treatment, p < 0.05 in both groups; however, no significant difference between groups have been retrieved*.

**** *Significant difference occurred in FMS assessed between baseline and post treatment, p < 0.001 in both groups; however, no significant difference between groups have been retrieved*.

***** *No significant difference occurred in FMS assessed between baseline and post treatment in both groups (p = 0.31 pre-PNS and p = 0.67 post-PNS); significant difference occurred for the SIS in the tDCS pre-PNS group (p = 0.02). However, no significant difference between groups have been retrieved for both FMS and SIS p = 0.59 and p = 0.07, respectively*.

****** *Significant interaction effect (p < 0.01) of treatment (real and sham-tDCS) and stroke location (subcortical and cortical)*.

§ *A significant interaction effect (p < 0.05) was detected regarding stroke duration (subacute vs. chronic) and type (cortical vs. subcortical)*.

**Table 2C:** **Clinical scales scores for Triccas et al. (2015)**.

	**FMS (SD)**	**ARAT (SD)**	**SIS (SD)**	**MAL (SD)**
Baseline	Subacute stroke: 36.7 ± 18.4 Chronic stroke: 27.55 ± 13.77	Subacute stroke: 33.5 ± 0.6 Chronic stroke: 6.0 ± 0.2	Subacute stroke: 58.0 ± 21.8 Chronic stroke: 58.1 ± 26.5	Subacute stroke: 1.3 ± 1.3 Chronic stroke: 0.5 ± 0.5
Post treatment^*^	Subacute stroke: 47.0 ± 17.8 Chronic stroke: 30.0 ± 10.23	Subacute stroke: 48.5 ± 0.6 Chronic stroke: 8.0 ± 0.2	Subacute stroke: 75.0 ± 15.7 Chronic stroke: 58.5 ± 23.4	Subacute stroke: 2.3 ± 1.8 Chronic stroke: 0.5 ± 0.7

* *Significant changes at post-intervention between stage (i.e., subacute vs. chronic) per time interaction have been retrieved*.

*FMS, Fugl-Meyer Score; ARAT, Action Research Arm Test; MAL, Motor Activity Log; SIS, Stroke Impact Scale; SD, Standard Deviation*.

### Timing effects of tDCS combined with robotic therapy

The temporal relationship between brain stimulation and robotic therapy may play an important role in the design of successful clinical protocols. The study in Edwards et al. (2009) showed that the increase of corticomotor excitability induced by a period of anodal tDCS was still present whether the brain stimulation was followed by robotic practice.

Following this approach, the study in Giacobbe et al. (2013) confirmed that significant improvements (differences between pre/post intervention) were retrieved only in movement smoothness when anodal tDCS is delivered before RT. Conversely, tDCS delivered during practice (aim indicator worsened of 15%) or after practice (a 10% significant speed decrease) seemed to offer no performance improvement (Table 3).

**Table 3:** **Kinematics indicators for Giacobbe et al. (2013)**.

		**Mean Speed (rad/s)**	**Peak Speed**	**Deviation (rad)**	**Smoothness**	**Duration (s)**	**Aim (rad)**
Expected trend		↑	↑	↓	↑	↓	↓
Sham tDCS during RT	Pre-training	3.1 ± 0.1 × 10^−1^ *p* = 0.006^**^	13.1 ± 0.8 × 10^−1^ *p* = 0.009^**^	3.6 ± 0.4 × 10^−2^ *p* = 0.168	2.8 ± 0.1 × 10^−1^ *p* = 0.317	3.74 ± 0.23 *p* = 0.432	7.9 ± 0.4 × 10^−1^ *p* = 0.095
	Post-training	3.7 ± 0.2 × 10^−1^ *p* = 0.006^**^	16.6 ± 1.1 × 10^−1^ *p* = 0.009^**^	4.4 ± 0.5 × 10^−2^ *p* = 0.168	2.6 ± 0.1 × 10^−1^ *p* = 0.317	3.48 ± 0.23 *p* = 0.432	8.8 ± 0.35 × 10^−1^ *p* = 0.095
tDCS before RT	Pre-training	3.65 ± 0.2 × 10^−1^ *p* = 0.43	16.4 ± 0.1 × 10^−1^ *p* = 0.297	4.7 ± 0.5 × 10^−2^ *p* = 0.133	2.5 ± 0.1 × 10^−1^ *p* = 0.001^**^	3.1 ± 0.3 *p* = 0.062	9.3 ± 0.3 × 10^−1^ *p* = 0.052
	Post-training	3.8 ± 0.2 × 10^−1^ *p* = 0.43	14.9 ± 0.1 × 10^−1^ *p* = 0.297	3.7 ± 0.4 × 10^−2^ *p* = 0.133	2.9 ± 0.1 × 10^−1^ *p* = 0.001^**^	3.2 ± 0.2 *p* = 0.062	8.3 ±0.4 × 10^−1^ *p* = 0.052
tDCS during RT	Pre-training	3.4 ± 0.2 × 10^−1^ *p* = 0.53	13.9 ±1 × 10-1 *p* = 0.585	3.6 ± 0.3 × 10^−2^ *p* = 0.239	2.8 ± 0.1 × 10^−1^ *p* = 0.554	3.01 ± 0.22 *p* = 0.987	7.1 ± 0.3 × 10^−1^ *p* = 0.019^*^
	Post-training	3.6 ± 0.2 × 10^−1^ *p* = 0.53	14.6 ± 0.9 × 10^−1^ *p* = 0.585	4.2 ± 0.5 × 10^−2^ *p* = 0.239	2.9 ± 0.1 × 10^−1^ *p* = 0.554	3.08 ± 0.21 *p* = 0.987	8.2 ± 0.3 × 10^−1^ *p* = 0.019^*^
tDCS after RT	Pre-training	4.1 ± 0.2 × 10^−1^ *p* = 0.032^*^	15.7 ± 0.9 × 10^−1^ *p* = 0.595	3.6 ± 0.4 × 10^−2^ *p* = 0.087	3.0 ± 0.1 × 10^−1^ *p* = 0.529	2.58 ± 0.18 *p* = 0.158	8.0 ± 0.4 × 10^−1^ *p* = 0.65
	Post-training	3.6 ± 0.2 × 10^−1^ *p* = 0.032^*^	15.0 ±1 × 10^−1^ *p* = 0.595	4.8 ± 0.5 × 10^−2^ *p* = 0.087	2.9 ± 0.1 × 10^−1^ *p* = 0.529	2.94 ± 0.19 *p* = 0.158	7.8 ± 0.3 × 10^−1^ *p* = 0.65

* *Significant REDUCTION in post intervention with respect to baseline (<20%)*.

** *Significant INCREASE in post intervention with respect to baseline (p < 0.05)*.

In addition, the results in Table 4 confirmed that motor practice has increased corticomotor excitability of the trained muscles while on the other hand, electrical stimulation delivered at any of the time points in relation to robotic training resulted in no significant changes in muscles MEP amplitude.

**Table 4:** **Neurophysiological indicators**.

**Study**	**Mean MEP (SD) Amplitude (FCR, ECR, muscles)**	Ipsilesional cortical map volume (normalized MEP amplitude^*^cm^2^) (mean)	**COG_x_ (cm) (lateral-medial)**	**COG_y_ (cm) (anterior-posterior)**
Giacobbe et al., 2013	Increased amplitude %FCR and ECR tDCS pre-RT (*n* = 6) (FCR) 97 ± 9% baseline (ECR) 124 ± 21% baseline tDCS during-RT (*n* = 5) (FCR) 139 ± 43% baseline (ECR) 115 ± 29% baseline tDCS post-RT (*n* = 5) (FCR) 110 ± 19% baseline (ECR) 103 ± 16% baseline	–	–	–
Powell et al., 2016^*^ (Baseline vs. post intervention)	–	Group A tDCS pre-PNS: 2.1 Group B tDCS post-PNS: −6.3	Group A tDCS pre-PNS: −0.62 (medial) Group B tDCS post-PNS: 0.3 (lateral)	Group A tDCS pre-PNS: 0.48 (anterior) Group B tDCS post-PNS: −0.48 (posterior)

* *Only one subject for each group (Powell et al., 2016). Ipsilesional map volume increased in Group A and decreased in Group B. COG location shifted in opposite directions according to stimulation condition. FCR, Flexor Carpi Radialis; ECR, Extensor Carpi Radialis; MEP, Motor Evoked Potential; COG_x_, ipsilesional Center of Gravity location change; COG_y_, ipsilesional Center of Gravity location change; SD, standard deviation*.

### Effects of anodal vs. cathodal tDCS combined with robotic practice

Another interesting aspect to be investigated is the different effect of anodal tDCS with respect to cathodal tDCS when delivered together with robotic therapy (Hesse et al., 2011; Ochi et al., 2013).

In Ochi et al. (2013) both interventions showed significant but moderate improvements in FMS and MAS in post intervention with respect to the baseline with a slight significant effect of cathodal tDCS in the MAS only for patients with right hemispheric lesions (Table 2A).

In addition, neither anodal nor cathodal tDCS enhanced the effects of bimanual RT (Hesse et al., 2011) on subacute stroke subjects in terms of FMS (Table 2A).

### Effects of anodal tDCS combined with other neurostimulation techniques and RT

In a recent study (Powell et al., 2016), timing variations of electrical brain stimulation paired with nerve stimulation and RT have been investigated to enhance motor recovery in chronic stroke subjects.

Slight significant effect has been observed for SIS in Group A while no significant changes have been retrieved in FMS for both groups and in SIS for Group B (Table 2B). Moreover, no between-group effects have been retrieved both for FMS and SIS.

Neurophysiological measurements were extracted only from two subjects; one subject in Group A showed an increase of cortical map volume while one subject in the Group B revealed a decrease for the same neurophysiological parameter (Table 4).

Another recent study (Ang et al., 2015) aimed to demonstrate the feasibility of using tDCS to facilitate the ability of stroke patients to operate a MI-BCI (Wolpaw et al., 2002) and, subsequently, the efficacy of the treatment together with robotic feedback in a sham-controlled randomized trial.

FMSs showed that real tDCS before MI-BCI and RT did not result in additional motor improvements compared to the single treatment (i.e., MI-BCI paired with robotic feedback) (Table 2B). On the other hand, the evaluation of online MI-BCI accuracy may have benefited from tDCS as already preliminary analyzed in Ang et al. (2012).

## Discussion

The analysis of the literature presented in this paper has strengthened the efficacy of RT in stroke rehabilitation (Lo et al., 2010; Hesse et al., 2011; Ochi et al., 2013; Klamroth-Marganska et al., 2014; Triccas et al., 2015; Straudi et al., 2016).

However, the following limitations regarding the effects of combining tDCS and robotic therapy, in motor and functional recovery were retrieved:

- Coupling unilateral (anodal or cathodal) or bilateral tDCS to different RT (unilateral or bilateral, distal or proximal) did not produce significant effects in terms of FMS with respect to RT alone either in chronic or subacute stroke patients (Hesse et al., 2011; Ochi et al., 2013; Triccas et al., 2015; Straudi et al., 2016);
- Delivering anodal tDCS during and after unilateral wrist RT did not increase kinematic performance in chronic stroke patients (Giacobbe et al., 2013);
- Chronic stroke subjects treated with tDCS at the end of PNS followed by RT did not improve their motor functions, as assessed by means of FMS and SIS (Powell et al., 2016).
- Administering tDCS before MI-BCI with robotic feedback did not enhance motor functions (assessed by FMS) in chronic stroke patients respect to MI-BCI treatment alone (Ang et al., 2015).

Nevertheless, despite these limitations, the reported studies show some encouraging findings that would deserve to be investigated by means of larger randomized controlled trials with standardized treatment protocols.

These findings are listed in the following:

– A single session of anodal tDCS during bilateral RT on subacute stroke patients leads to significant improvements in FMS (Hesse et al., 2007);
– Delivering tDCS before RT seems to be more effective than during or after RT (Giacobbe et al., 2013);
– tDCS may enhance the averaged accuracy of classifying the MI of the stroke-affected upper limb (Ang et al., 2015).
– Combined with RT, cathodal stimulation of the contralateral hemisphere could yield higher effects with respect to anodal tDCS stimulation of the affected hemisphere considering lesion side (Ochi et al., 2013);
– Slight clinical effects of anodal tDCS plus exoskeletal robotic treatment were found in subacute vs. chronic stroke patients (Triccas et al., 2015);
– Bilateral tDCS combined with proximal upper limb RT seems to be more effective in chronic patients with subcortical lesions (Straudi et al., 2016);
– Delivering excitatory tDCS before PNS and RT may enhance the functional outcomes of chronic stroke patients more than applying tDCS after PNS and before RT (Powell et al., 2016).

The large variability in the characteristics of patients enrolled in different studies (chronic or acute, ischemic or hemorrhagic, cortical or subcortical lesion) and the lack of a standardized intervention protocol make difficult to compare and provide a definitive analysis of the results.

However, an attempt of analyzing factors influencing outcomes herein presented has been carried out. Several factors have been identified and discussed as responsible for the large variety of the reported results. They are discussed in the following subsections.

### Several types of intervention and treatment intensity

Type of intervention and treatment intensity may represent crucial aspects for evaluating efficacy of tDCS coupled with robot-aided rehabilitation. In fact, from the literature analysis emerges that tDCS and RT effects have been investigated in multiple manners, thus producing different results.

For instance, the feasibility of the combined approach (tDCS and RT) both in chronic and in subacute stroke patients (Hesse et al., 2007; Giacobbe et al., 2013) has been studied in a single-session protocol without a control group.

Moreover, unilateral and/or bilateral tDCS has been applied without considering that the patients characteristics (i.e., type of stroke, lesion site, time post stroke) might have influenced the current findings.

Another important factor to be considered is the delivering time of tDCS respect to RT as well as the inhibitory and/or excitatory approach. In fact, duration, frequency, timing, and polarization of the stimulation are different in every study.

Furthermore, the use of different techniques such as PNS (Powell et al., 2016) and MI-BCI (Ang et al., 2015) in adjunct to tDCS and RT might also have influenced final FMS outcomes (Ang et al., 2015) either enhancing or decreasing the neuromodulatory effect.

For example, PNS activates proprioceptive sensory fibers (Kaelin-Lang et al., 2002) that results in increased excitability of motor cortex (Ridding et al., 2000); in this condition applying tDCS before, during or after PNS may result in constructive or disruptive effects in the targeted motor cortex area.

Finally, the use of tDCS to promote better operation of MI-BCI by stroke subjects represents a new research field. The detection of event-related desynchronization or synchronization (ERD/ERS) (Pfurtscheller and Da Silva, 1999) with EEG-motor imagery is expected to be enhanced by excitatory effect of anodal tDCS (Ang et al., 2012). Anodal tDCS may be employed as a conditioning tool for BCI in stroke (Kasashima et al., 2012), thus increasing the online accuracy of MI-BCI performance.

Stimulation parameters, such as electrical current intensity, represent the other factor that might have influenced results of the different studies; current stimulation varies in between 1 and 2 mA for different durations in the analyzed studies. So far, whether choosing different currents is equally effective in terms of brain stimulation remains unknown (Triccas et al., 2016).

Moreover, in all the selected studies tDCS was delivered using two common large electrodes; recent developments suggest the use of multiple small electrodes that allow optimizing the applied currents to achieve effective and targeted stimulation while ensuring safety of stimulation. Such an aspect may lead to tailor the stimulation to specific patients' population (Dmochowski et al., 2011).

### Different primary outcomes

FMS is the primary outcome for all the selected studies, except for Giacobbe et al. (2013) where kinematics and neurophysiological parameters are adopted as reference measurements for evaluating motor recovery.

It can be observed that the FMS improvements between pre/post treatment for all the different protocols do not seem to show benefit deriving from the adjunct of tDCS both in real, cathodal, or bilateral configuration. On the other hand, the reported findings strengthen the efficacy of RT in stroke rehabilitation. Furthermore, the analysis of some neurophysiological measurements (Ang et al., 2015; Powell et al., 2016) revealed that tDCS can provide either a benefit or an obstructive effect on chronic stroke subjects when is coupled with other techniques (MI-BCI and PNS) and robotic therapy, depending on the type, size, and location of the lesion.

### Type of stroke and lesion site

Another factor that makes difficult to perform a comparative analysis among the studies is the different cohort of recruited patients and the non-homogeneity in their residual abilities.

Depth of the brain lesion, the lesion side, and stroke duration are crucial parameters in determining the more effective rehabilitative treatment. From the analysis of the literature, it emerges that chronic and subacute patients respond in different manners to the same intervention; in fact, subacute subjects undergoing anodal tDCS and RT may have greater improvements with respect to chronic stroke patients (Triccas et al., 2015). One possible explanation is that subacute patients may take advantage from the spontaneous natural recovery that normally occurs during the first days after stroke onset.

The location of the lesion is also another important discriminating factor; in fact, chronic stroke patients with subcortical lesion seem to improve better than subacute with cortical damage when treated with the combined approach (Hesse et al., 2011; Straudi et al., 2016). Patients with subcortical lesion may find more benefit respect to patients with cortical lesion since their cortical connectivity remains intact after a stroke.

### Different robotic treatment

The last factor that may have influenced effects of the combined approach is represented by the different robotic treatments that are delivered, i.e., bilateral vs. unilateral arm training as well as planar vs. three-dimensional movements.

Some previous studies (Lin et al., 2008; Waller et al., 2008; Wu et al., 2011; Mazzoleni et al., 2013) have investigated effects of bilateral robotic arm training (BRT) vs. unilateral robotic arm training (URT). They have shown that URT has produced greater functional gains together with an increased use of the paretic arm in daily life, whereas BRT has improved proximal upper limb motor function and force generation or movement smoothness.

However, although the BiManu Track, InMotion2. and InMotion3 are conceived for different training methodologies, no great differences in terms of task difficulty are retrieved.

On the other hand, the Armeo® Spring and the ReoGo™ enable to actively train the entire arm thus indirectly allowing patients to compensate hand movements with shoulder. All these characteristics, combined with electrical stimulation may have influenced the Fugl-Meyer total scores at the end of treatment.

### Final considerations

It is worth considering that all the attempted approaches of neuromodulation are based on the inter-hemispheric inhibitory competition model. However, whether “up-regulation” of lesioned hemisphere lead to better results than “down-regulation” of contralateral hemisphere may not be valid in all conditions and for all patients. In fact, depending on the size of the lesion and the gravity of the impairment (moderate-to-severe impairment) it might be preferable to use the excitatory approach rather the inhibitory.

Recent findings suggest a possible alternative approach of neuromodulation. This method is based on the inhibition of the ipsilesional motor cortex aimed to favor motor learning through mechanisms of “homeostatic” plasticity (Di Lazzaro et al., 2013).

Recently, Di Pino et al. (2014) proposed a bimodal balance-recovery model, in which the influence of inter-hemispheric balancing on functional recovery depends on the structural reserve spared by the lesion. In this light, the above-described sources of individual variability, including lesion location and size as well as impaired connectivity, are all factors that could contribute to determine the theorized concept of structural reserve and thus guide the neuromodulatory intervention toward a “*rebalancing*” or a “*vicariation*” approach.

In this context, a multimodal pre-therapy diagnosis could be envisaged, involving the clinical history of the patient, time elapsed after the stroke, topography of the lesion, and type and severity of functional impairment (Di Pino et al., 2014).

Such a patient-tailored approach together with technology advancements in robotic devices may lead to obtain better improvements in motor and functional recovery.

## Conclusions and future perspectives

This paper has presented a literature analysis of combining non-invasive electrical brain stimulation (tDCS) with upper limb robotic therapy. The large variability in the characteristics of patients enrolled in different studies (chronic or acute, ischemic or hemorrhagic, cortical or subcortical lesion) and the lack of a standardized intervention protocol make difficult to reach a definite conclusion.

Restoration of motor control seems to be the same after electrical brain stimulation coupled with RT and after RT alone as supported by a quantitative analysis of FMSs.

Furthermore, these findings confirm the effectiveness of RT in post-stroke patients.

However, slight encouraging effects arise accounting for the lesion site, the type of stroke and type of damage thus suggesting the development of patient-tailored rehabilitative treatments.

In future studies, a more individually tailored approach is required to choose the best type of tDCS (excitatory and/or inhibitory) and the best possible target (ipsilesional or contralesional hemisphere) after taking into consideration several parameters. Moreover, such studies will help to refine the bimodal balance–recovery model and test whether the efficacy of individualized NIBS therapy combined with robotic therapy might result superior to the current methods.

## Author contributions

DS and LZ designed the paper, analyzed the literature, analyzed robot data, and wrote the manuscript. StM partly analyzed the clinical literature together with SaM, MB, and GM and they also revised the manuscripts. FR revised the manuscript and contributed to write clinical parts related to neuromodulation. EG, VD, and SS supervised the writing. All the authors read and approved the final version of the manuscript.

### Conflict of interest statement

The authors declare that the research was conducted in the absence of any commercial or financial relationships that could be construed as a potential conflict of interest. The reviewer PL and handling Editor declared their shared affiliation, and the handling Editor states that the process nevertheless met the standards of a fair and objective review.
